# The utility of the prehospital shock index, age shock index, and modified shock index for predicting hypofibrinogenaemia in trauma patients: an observational retrospective study

**DOI:** 10.1007/s00068-024-02603-x

**Published:** 2024-08-07

**Authors:** Jihwan Moon, Sungwook Park

**Affiliations:** Department of Emergency Medicine, Pusan National University Hospital, 179, Gudeok-ro, Seo- Gu, Busan, 49241 Republic of Korea

**Keywords:** Fibrinogen, Hemorrhage, Shock index, Trauma

## Abstract

**Purpose:**

Reduced fibrinogen levels are associated with worse outcomes in bleeding trauma patients. The purpose of this study was to evaluate the potential of the prehospital shock index (SI) and its derivatives, the age shock index (aSI) and the modified shock index (mSI), as predictors of hypofibrinogenaemia in trauma patients.

**Methods:**

This retrospective study included 2383 patients who presented to a regional trauma center. We reviewed the plasma fibrinogen levels upon admission to the trauma center and patients were divided into two groups: the hypofibrinogenaemia group and the normal group. The predictive performances of the SI, aSI, and mSI were assessed by the area under the receiver operating characteristic curve (AUC).

**Results:**

Of the 2383 patients, 235 (9.9%) had hypofibrinogenaemia. Patients with hypofibrinogenaemia were more likely to receive transfusions within 4 h and had significantly greater in-hospital mortality than patients with normal fibrinogen levels. The AUCs of prehospital SI, prehospital aSI, and prehospital mSI for the prediction of hypofibrinogenaemia were 0.75 (95% confidence interval [CI] 0.73–0.77), 0.70 (95% CI 0.68–0.72), and 0.75 (95% CI 0.73–0.77), respectively.

**Conclusion:**

Prehospital SI and prehospital mSI demonstrated moderate performance for identifying trauma patients with hypofibrinogenaemia. The prehospital aSI had poor predictive performance. In the prehospital setting, the use of prehospital SI or prehospital mSI as the sole predictor of hypofibrinogenaemia in trauma patients is not recommended.

## Introduction

Trauma is a leading cause of morbidity and mortality worldwide, and severe trauma often leads to critical complications such as trauma-induced coagulopathy (TIC). TIC represents a significant challenge in the management of severely injured patients, affecting approximately 25% of patients upon hospital admission [1].

TIC manifests with fibrinogen depletion as the initial abnormal coagulation parameter, and plasma fibrinogen levels reach critically low levels earlier and more frequently than standard coagulation parameters [2–4]. Several studies have shown that a low admission fibrinogen level is an independent predictor of the need for massive transfusion [5, 6] and increased mortality [7–11] in trauma patients. For coagulation support, current guidelines recommend that fibrinogen supplementation should begin in the presence of plasma fibrinogen levels ≤ 150 mg/dL or viscoelastic signs of fibrinogen deficiency [12]. However, a total of 61% of countries, including South Asia, have blood shortages, resulting in trauma patients in these countries not being guaranteed immediate access to transfusions [13]. In South Korea, access to comprehensive coagulation diagnostics, including viscoelastic tests for fibrinogen deficiency, and emergency transfusions and fibrinogen supplementation are available only at large university hospitals or high-level trauma centers. Therefore, if patients without obvious signs of relevant bleeding but at high risk for hypofibrinogenaemia are undertriaged to lower-level medical facilities, they may not receive appropriate treatment for coagulopathy. Given these challenges, early identification of patients at risk for hypofibrinogenaemia and their prompt triage to specialized trauma centers equipped to manage such cases effectively is crucial.

The shock index (SI) and its derivatives, including the age shock index (aSI) and modified shock index (mSI), which are readily available and widely used in prehospital settings, reflect the physiological response to hypovolemia in hemorrhage patients. However, few studies have examined the association between the shock index and fibrinogen levels [14]. Therefore, this retrospective observational study aimed to determine whether prehospital SI and its derivatives can predict low plasma fibrinogen levels in trauma patients. We hypothesized that the SI, aSI, and mSI obtained in the prehospital setting may have the potential to predict patients with low plasma fibrinogen levels upon admission.

## Methods

### Study design and patients

This retrospective observational study was conducted at the trauma center of a 1,400-bed tertiary care university-affiliated hospital in Busan, Korea. Functioning as a Level I regional trauma center, the facility offers specialized care primarily to patients from Busan city and Kyung-Nam Province. All consecutive patients aged 16 years and older who presented to the trauma center between January 1, 2016, and December 31, 2022, were included. The exclusion criteria were patients who experienced cardiac arrest before or were dead on arrival at the emergency department (ED), patients aged less than 16 years, patients transferred from other hospitals, patients with missing prehospital vital signs (systolic blood pressure [SBP], diastolic blood pressure [DBP], or heart rate [HR]), and those with no admission plasma fibrinogen levels. Prior to the initiation of the study, we obtained approval from the hospital’s institutional review board (IRB) (approval number 2401-006-187). Informed consent was waived in accordance with the IRB regulations.

### Data collection

The Korean Trauma Database (KTDB), maintained by the Ministry of Health and Welfare of Korea, aims to collect and compile clinical information on trauma patients. It aggregates the national trauma registry, which is a specialized data collection consisting of a set of standardized elements, including details on the injury event, prehospital information, demographics, diagnosis, and treatment outcomes for injured patients. From the KTDB registry, we collected data on age, sex, mechanism of injury, time from injury to trauma center arrival, time between emergency medical service (EMS) departure from the scene and trauma center arrival, prehospital fluid administration, prehospital vital signs (SBP, DBP, and HR), Injury Severity Score (ISS), total Glasgow Coma Scale (GCS) score, incidence of blood product administration within 4 h after arrival at the ED, intensive care unit admission, and in-hospital mortality. Laboratory values (hemoglobin [Hb], platelet [PLT], prothrombin time international normalized ratio [PT INR], and ethanol) and arterial blood gas analysis (ABGA) findings (pH, base excess [BE], and lactate) were obtained from the electronic medical records of each patient.

### Blood sample collection

Routine arterial and venous blood samples were collected simultaneously and immediately upon the patient’s arrival at the trauma center. Laboratory variables were measured using venous blood samples, and fibrinogen levels were analyzed by the Clauss assay. Arterial blood samples were used for ABGA, which was immediately performed using the GEM Premier 3500 system (Instrumentation Laboratory, Massachusetts, USA) kept in the ED.

### Definition

Hypofibrinogenaemia was defined as a plasma fibrinogen concentration < 150 mg/dL.

Prehospital SI was defined as the ratio of prehospital HR to SBP.

Prehospital aSI was defined as age multiplied by the prehospital SI.

Prehospital mSI was defined as the ratio of prehospital HR to MAP (mean arterial pressure = [2 × DBP + SBP] ÷ 3).

### Statistical analysis

Continuous variables were presented as means and standard deviations (SDs) after testing for a normal distribution using the Kolmogorov-Smirnov test. Categorical variables were shown as absolute and relative frequencies. Variables between groups with hypofibrinogenaemia and normal fibrinogen levels were compared using Student’s t- test for continuous variables and the chi-squared test or Fisher’s exact test for categorical variables. Each index was entered into a logistic regression analysis to evaluate the individual risk of hypofibrinogenaemia. Variables with a p-value of < 0.05 in the univariate analysis were included in the logistic regression model. Therefore, time between EMS departure from scene and trauma center arrival, prehospital fluid administration (yes/no), GCS, hemoglobin, platelet, PT INR, pH, lactate, and base excess were selected as covariates. We performed the logistic regression analysis by using eacn index as binary variable based on optimal cut-off values. Additionally, we performed the logistic regression analysis with prehospital SI, aSI and mSI standardized to have a mean of 0 and a SD of 1. We used a backward elimination method. The results from the logistic regression analysis were summarized using odds ratios (ORs) and their respective 95% confidence intervals (CIs). To evaluate the predictive performance, receiver operating characteristic (ROC) curves were constructed and the area under the curve (AUC) was calculated. To determine an optimal cut-off value for each index to differentiate patients with hypofibrinogenaemia from those with normal plasma fibrinogen levels, we chose the cut-off point with the maximum Youden index (Youden index: sensitivity + specificity − 1). In addition, we evaluated the sensitivity, specificity, positive likelihood ratio (PLR), negative likelihood ratio (NLR), positive predictive value (PPV), and negative predictive value (NPV) for predicting hypofibrinogenaemia at the cut-off values derived from each index. A two-tailed p-value of < 0.05 was considered significant. All the statistical analyses were conducted using IBM SPSS Statistics for Windows, version 21.0 (IBM Corp., Armonk, NY, USA) and MedCalc for Windows, version 22.023 (MedCalc Software Ltd, Ostend, Belgium).

## Results

### The characteristics of the total study patients

**Fig. 1:**
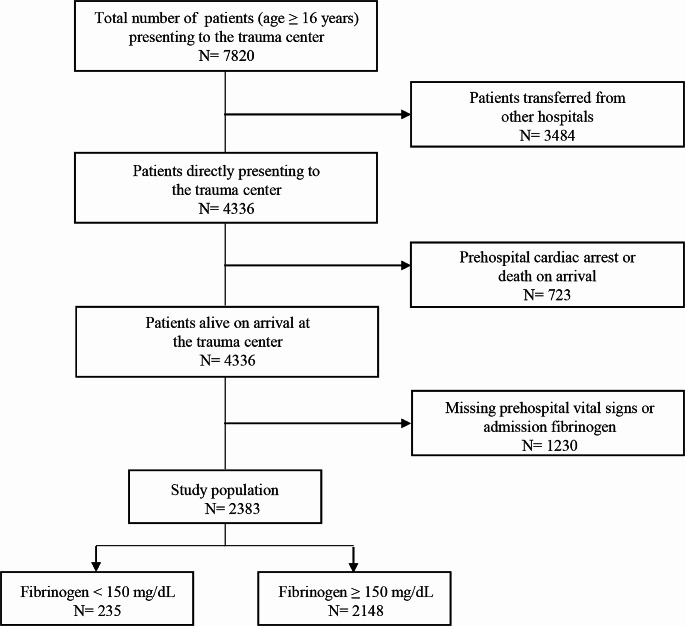
Flow chart of patient inclusion

A total of 7820 patients aged 16 years and older were admitted to our trauma center between January 1, 2016, and December 31, 2022. Of those, 3484 patients were excluded from the study because they were transferred from other hospitals, 723 patients experienced cardiac arrest before or upon arrival at the trauma center, and 1230 patients had no available admission fibrinogen level or prehospital vital signs. Thus, the remaining 2383 patients composed our study group. Figure 1 details the flow of patients throughout the study. Among the 2383 patients, 77.0% were male, and the mean age was 55.2 ± 18.1 years. The mean ISS was 19.9 ± 11.9, and the mean admission fibrinogen level was 237 ± 72 mg/dL. A total of 39.7% of the study patients received transfusions within 4 h of admission, and the in-hospital mortality rate was 11.4%.

### Clinical outcomes

A total of 235 patients developed hypofibrinogenaemia, and these patients were included in the hypofibrinogenaemia group. The other 2,148 patients who had fibrinogen levels ≥ 150 mg/dL were included in the normal group. Table 1 shows the demographic data and clinical characteristics of the patients. In the comparative analysis between the two groups, significant differences were observed in time between EMS departure from the scene and arrival at the trauma center, prehospital fluid administration, SBP, DBP, HR, prehospital SI, prehospital aSI, prehospital mSI, Hb, PLT, PT INR, ethanol, pH, BE, lactate, ISS, transfusion within 4 h, ICU admission, and in-hospital mortality (all p-values were less than 0.001 except for ethanol [*p* = 0.019]). There were no significant differences between the two groups in terms of age, sex, mechanism of injury, and time from injury to arrival at the trauma center. After adjusting for covariates, all indices were still associated with hypofibrinogenaemia. When each index was used as binary variable, logistic regression analysis showed the following results: prehospital SI (OR 10.6, 95% CI = 7.3–15.2, *p* < 0.001), prehospital aSI (OR 4.6, 95% CI = 3.1–6.7, *p* < 0.001), and prehospital mSI (OR 8.8, 95% CI = 6.0-12.9, *p* < 0.001). When each index was standardized to have a mean of 0 and a SD of 1, the results were as follows: prehospital SI (OR 3.4, 95% CI = 2.8–4.1, *p* < 0.001), prehospital aSI (OR 1.9, 95% CI = 1.6–2.2, *p* < 0.001), and prehospital mSI (OR 3.2, 95% CI = 2.7–3.9, *p* < 0.001) (Table 2).

**Table 1:** Baseline characteristics of the enrolled patients

	Hypofibrinogenaemia (*n* = 235)		Normal (*n* = 2148)	*p* -value
Age, years	54.6 ± 19.9	55.3 ± 17.9		0.655
Age ≥ 65 years, n (%)	75 (31.9)	682 (31.8)		0.959
Males, n (%)	183 (77.9)	1653 (77.0)		0.807
Mechanism of injury, n (%)				0.058
Traffic accident	123 (52.3)	1076 (50.1)		
Fall down	75 (31.9)	619 (28.8)		
Slip down	5 (2.1)	53 (2.5)		
Struck by object	7 (3.0)	122 (5.7)		
Cutting or piercing	12 (5.1)	203 (9.5)		
Others	13 (5.5)	75 (3.5)		
Time from injury to TC arrival, (min)	52.3 ± 38.7	54.7 ± 53.0		0.509
Time between EMS departure from scene and TC arrival, (min)	24.9 ± 14.9	27.4 ± 18.9		< 0.001
Prehospital fluid administration (yes), n (%)	46 (19.6)	136 (6.3)		< 0.001
Prehospital vital sign				
SBP (mmHg)	97.9 ± 44.6	129.5 ± 25.1		< 0.001
DBP (mmHg)	56.8 ± 26.7	76.9 ± 16.7		< 0.001
HR (beats/min)	99.5 ± 23.9	91.5 ± 21.5		< 0.001
Shock index	1.2 ± 0.6	0.7 ± 0.2		< 0.001
Age shock index	64.9 ± 39.2	39.2 ± 15.5		< 0.001
Modified shock index	1.7 ± 0.8	1.0 ± 0.3		< 0.001
TC admission				
Total Glasgow Coma Scale score	9.7 ± 4.9	12.6 ± 3.8		< 0.001
Hemoglobin (g/dl)	12.1 ± 2.3	13.5 ± 1.9		< 0.001
Platelet (×10^3^/µl)	201.6 ± 75.5	249.8 ± 67.8		< 0.001
PT INR	1.4 ± 0.8	1.1 ± 0.1		< 0.001
pH	7.34 ± 0.12	7.40 ± 0.09		< 0.001
Lactate (mmol/l)	5.1 ± 3.3	3.3 ± 2.4		< 0.001
Base excess (mEq/l)	-4.5 ± 6.1	-1.2 ± 4.9		< 0.001
Ethanol (mg/dl)	85.8 ± 113.5	67.1 ± 101.9		0.019
Results of trauma				
ISS	26.8 ± 10.8.	19.1 ± 11.8		< 0.001
ISS ≥ 16, n (%)	204 (86.8)	1275 (59.4)		< 0.001
Transfusion within 4 h, n (%)	185 (78.7)	762 (35.5)		< 0.001
ICU admission, n (%)	219 (93.2)	1576 (73.4)		< 0.001
In-hospital mortality, n (%)	93 (39.6)	178 (8.3)		< 0.001

EMS, emergency medical service; TC, trauma center; SBP, systolic blood pressure; DBP, diastolic blood pressure; HR, heart rate; PT INR, prothrombin time international normalized ratio; ISS, injury severity score; ICU, intensive care unit

**Table 2:** Logistic regression analysis results for the association of prehospital shock indices with hypofibriongenaemia

	Binary^*^				Standardized^*^	
	OR (95% CI)	p			OR (95% CI)	p
Prehospital SI	10.6 (7.3–15.2)	< 0.001		Prehospital SI (1 SD)	3.4 (2.8–4.1)	< 0.001
Prehospital aSI	4.6 (3.1–6.7)	< 0.001		Prehospital aSI (1 SD)	1.9 (1.6–2.2)	< 0.001
Prehospital mSI	8.8 (6.0-12.9)	< 0.001		Prehospital mSI (1 SD)	3.2 (2.7–3.9)	< 0.001

OR, odds ratio; CI, confidence inerval; SI, shock index; aSI, age shock index; mSI, modified shock index; SD, standardard deviation

^*^ The analysis was performed using two approaches: with each index as a binary variable based on optimal cut off values, and with each index standardized to have a mean of 0 and a SD of 1

The respective cut-off values for prehospital SI, prehospital aSI, and prehospital mSI are 1.03, 63.5, and 1.43

The respective SDs for prehospital SI, prehospital aSI, and prehospital mSI are 0.32, 20.83, and 0.45

Each index was adjusted for time between emergency medical service departure from scene and trauma center arrival, prehospital fluid administration (yes/no), Total Glagow Coma Scale score, hemoglobin, platelet, prothrombin time international normalized ratio, pH, lactate, and base excess

### Performance of prehospital SI and its derivatives in predicting hypofibrinogenaemia

**Fig. 2:**
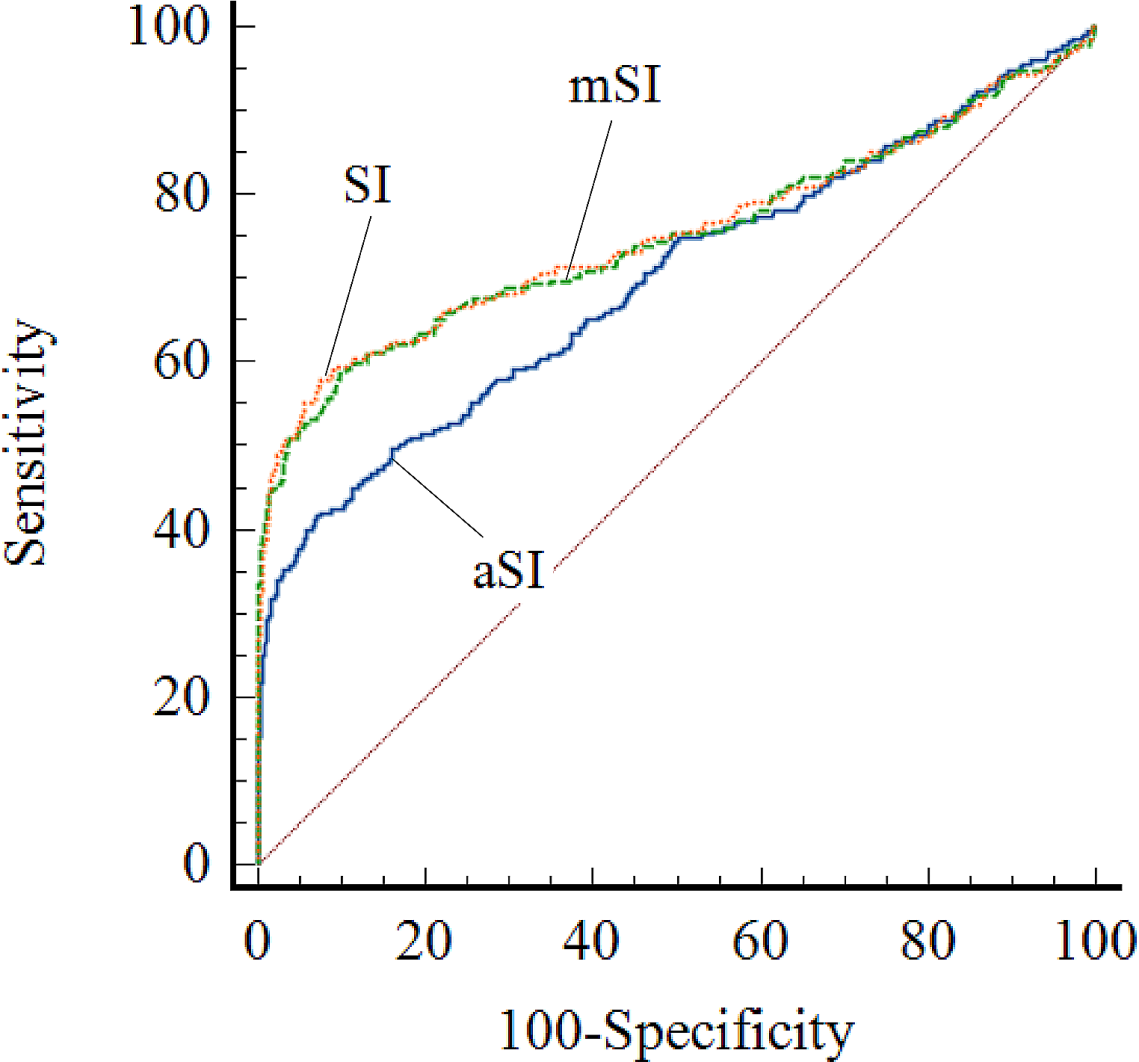
Performance of the prehospital shock index, prehospital age shock inedx, and prehospital modified shock index in predicting hypofibrinogenaemia

The AUCs of prehospital SI, prehospital aSI, and prehospital mSI for the prediction of hypofibrinogenaemia were 0.75 (95% CI 0.74–0.77), 0.70 (95% CI 0.68–0.72), and 0.75 (95% CI 0.73–0.77), respectively. The overall AUC values of prehospital SI were similar to those of prehospital mSI, and both were greater than that of prehospital aSI (Fig. 2). The cutoff values for the prehospital SI, prehospital aSI, and prehospital mSI for predicting hypofibrinogenaemia were 1.03, with a sensitivity of 59.6% and a specificity of 90.9%; 63.51, with a sensitivity of 41.7% and a specificity of 93.0%; and 1.43, with a sensitivity of 58.7% and a specificity of 90.1%, respectively. The PPV and NPV were 40.9% and 95.3% for prehospital SI, and 39.3% and 95.2% for prehospital mSI, respectively. The PLR and NLR were 6.56 and 0.44 for prehospital SI, and 5.53 and 0.46 for prehospital mSI, respectively (Table 3).

**Table 3:** Peformance of prehospital shock index, prehospital age shock index, and prehospital modified shock index for predicting hypofibrinogenaemia

	Cut-off	Se (%)	Sp (%)	PPV (%)	NPV (%)	PLR	NLR
Prehospital SI	1.03	59.6	90.9	40.9	95.3	6.56	0.44
Prehospital aSI	63.5	41.7	93.0	39.5	93.6	5.56	0.63
Prehospital mSI	1.43	58.7	90.1	39.3	95.2	5.53	0.46

SI, shock index; aSI, age shock index; mSI, modified shock index; Se, sensitivity; Sp, specificity; PPV, positive predictive value; NPV, negative predictive value; PLR, positive likelihood ratio; NLR, negative likelihood ratio

## Discussion

This retrospective observational study aimed to determine whether prehospital SI, prehospital aSI, and prehospital mSI can reliably predict low fibrinogen levels on admission in trauma patients. Our results showed that prehospital SI and prehospital mSI had moderate accuracy, while prehospital aSI had poor accuracy in predicting patients with hypofibrinogenaemia on admission.

Fibrinogen, the precursor to fibrin, is essential for stable clot formation. After major trauma hemorrhage, it becomes depleted due to a combination of fibrinogen consumption, fibrinolysis, and dilution [4, 15]. One prospective cohort study has shown that low admission fibrinogen level is independently associated with increased injury severity and shock [10]. This finding was also confirmed in our study. In our study, patients with hypofibrinogenemia had lower prehospital blood pressure, higher injury severity scores, and received more blood transfusions within the first four hours of admission compared to patients with normal fibrinogen levels. Additionally, ICU admission rates and in-hospital mortality were significantly higher in patients with hypofibrinogenemia. Our study showed that prehospital SI, prehospital aSI and prehospital mSI were independent predictors of hypofibrinogenaemia. These highlight that in patients with prehospital SI above cut-off values, testing for plasma fibrinogen levels or conducting viscoelastic tests should be considered.

Several studies have identified the predictors of hypofibrinogenaemia in trauma patients [16–18]. In these studies, age, sex, prehospital fluid administration, scores such as the Triage Revised Trauma Score or ISS, and laboratory variables such as pH and BE were significantly associated with hypofibrinogenaemia on admission. However, these variables are difficult to apply in the prehospital setting due to the reliance on laboratory values obtained in the ED, and the scoring systems are complex and difficult to calculate.

Upon reviewing the literature, only one study examined the utility of the shock index in predicting hypofibrinogenemia in trauma patients. Škola et al. conducted a prospective observational study on trauma patients admitted from helicopter EMS to two large trauma centers and reported that the worst prehospital SI predicted hypofibrinogenemia with an AUC of 0.79 (95% CI 0.64–0.91) and that the admission SI had an AUC of 0.79 (95% CI 0.66–0.91) [14]. In this study, a prehospital SI ≥ 1 had a sensitivity of 0.5 (95% CI 0.19–0.81), a specificity of 0.88 (95% CI 0.83–0.92), and an NPV of 0.98 (95% CI 0.96–0.99). These findings are in line with the results of our study. Based on the AUC of this study and our study, the prehospital SI and prehospital mSI appear to have moderate accuracy in identifying patients with hypofibrinogenemia. To further evaluate the usefulness of prehospital SI and prehospital mSI, we calculated the posttest probability of hypofibrinogenemia. Because the exact prevalence of hypofibrinogenemia in trauma patients is not well established, we assumed that the pretest probability of hypofibrinogenemia was 25%, based on the prevalence of TIC [1]. Above the cut-off values of prehospital SI and prehospital mSI (with PLR = 6.56, 5.53, respectively) produced posttest probabilities of 68.4% and 64.6%, respectively. This means that transporting all patients with prehospital SI or prehospital mSI above the cut-off value to higher-level trauma centers for hypofibrinogenemia testing and treatment may result in overtriage rates exceeding 30%. To reduce the high false positive rate for hypofibrinogenemia, the cut-off value for prehospital SI or prehospital mSI could be set higher. However, while this would increase the PLR and decrease the false positive rate, it would also result in fewer patients meeting the higher cut-off value, leading to decreased clinical efficacy of both indices. Conversely, below the cut-off values of prehospital SI and prehospital mSI (with NLR = 0.44, 0.46, respectively) yielded posttest probabilities of 12.7% and 13.2%, respectively. This means that if all patients with a SI or mSI below the cutoff value are transported to lower-level trauma centers, approximately 13% of them will be undertriaged. Overall, these posttest probabilities based on the prehospital SI and prehospital mSI may not be acceptably high or low enough to reliably identify patients with or without hypofibrinogenemia (Table 4). Furthermore, in the CRYOSTAT-2 randomized clinical trial, the addition of early and empirical high-dose cryoprecipitate to standard care for fibrinogen replacement did not improve all-cause 28-day mortality in patients who had evidence of active hemorrhage and a SBP of less than 90 mm Hg at any time [19]. Therefore, relying solely on physiological parameters such as the prehospital shock index to predict hypofibrinogenemia or to guide fibrinogen supplementation is not recommended. Instead, fibrinogen supplementation for hypofibrinogenemia should be guided by laboratory values.

**Table 4:** Probabilities of hypofibrinogenaemia after positive and negative results for prehospital shock index, prehospital age shock index, and prehospital modified shock index

	Prehospital SI	Prehospital aSI	Prehospital mSI
Pretest probability^*^	25%	25%	25%
Positive test probability (If a patient’s index is above the cut-off value)	68.4%	64.7%	64.6%
Negative test probability (If a patient’s index is below the cut-off value)	12.7%	17.2%	13.2%

SI, shock index; aSI, age shock index; mSI, modified shock index

^*^Pretest probability: set at 25% based on the prevalence of trauma induced coagulopathy [1]

In this study, age was not significantly different between patients with normal fibrinogen levels and those with hypofibrinogenaemia, and the predictive ability of the prehospital aSI for hypofibrinogenaemia was lower than that of the prehospital SI and prehospital mSI. These results were not consistent with those of previous studies, which revealed that hypofibrinogenaemia on admission was more common in younger patients [16, 20]. These discrepancies between our results and those of previous studies may be attributed to differences in the study populations. In the study by Gauss et al. [20], the mean age of the subjects was 33 years, which was significantly lower than the mean age of 55 years in our study. Additionally, the study by Kimura et al. [16] included a total of 290 patients, which is significantly smaller than our study population. Research on the relationship between age and fibrinogen levels in trauma patients is scarce, leaving the association between age and fibrinogen levels unclear.

This study has several limitations. First, it is important to acknowledge that this study was a retrospective analysis, thus there may be potential biases and confounding factors that could influence our results, as frequently mentioned in the literature. To address these limitations, a prospective, validated study is needed. Second, our study was conducted at a single regional trauma center, and the majority of the study population consisted of major trauma patients. Therefore, the results of this study cannot be generalized to other institutions or patient populations. Third, information about patients’ medications, such as antihypertensive drugs, analgesics, and drugs for depression, was not collected. Additionally, the preexisting comorbidities of the patients were not analyzed. Therefore, the impact of patients’ medications and comorbidities on BP, HR, and plasma fibrinogen levels was not considered. Fourth, there was a reduction in the overall study population size due to the presence of missing data, such as prehospital BP or HR. Patients without a recorded BP or HR might have had blood pressure too low to be measured, resulting in missing records. These patients are likely to have hypofibrinogenaemia. The inability to measure blood pressure is an inherent limitation of prehospital SI. Nonetheless, if these patients had been included in the study, the incidence of hypofibrinogenaemia might have been greater, potentially improving the sensitivity and specificity of the findings. Lastly, fluids were more frequently administered to patients with hypofibrinogenaemia than to patients with normal fibrinogen levels. Therefore, it cannot be ruled out that the prehospital administration of fluids may have affected the fibrinogen levels of the patients with hypofibrinogenaemia. However, the difference in the frequency of fluid administration was not large, at 13.3%, and the average time from the scene to arrival at the trauma center was 24.9 min, which is a very short time for fluid administration. Thus, the impact of fluid administration in the prehospital stage on fibrinogen levels would be very limited.

## Conclusion

Our study demonstrated that prehospital SI and prehospital mSI may have a limited role in identifying trauma patients with hypofibrinogenaemia. The prehospital aSI had poor predictive performance. Using prehospital SI or prehospital mSI as the sole predictor is not recommended when predicting hypofibrinogenaemia in trauma patients.

## Data Availability

Since the Ministry of Health and Welfare of Korea owns the data, they cannot be shared with public. Data are made available to researchers who satisfy the requirements for access to private information after the letter requesting access is reviewed.

## References

[1] Brohi K, Singh J, Heron M, Coats T. Acute traumatic coagulopathy. J Trauma. 2003;54:1127–30. 12813333 10.1097/01.TA.0000069184.82147.06

[2] Hayakawa M, Gando S, Ono Y, Wada T, Yanagida Y, Sawamura A. Fibrinogen level deteriorates before other routine coagulation parameters and massive transfusion in the early phase of severe trauma: a retrospective observational study. Semin Thromb Hemost. 2015;41:35–42. 25590522 10.1055/s-0034-1398379

[3] Hiippala ST, Myllylä GJ, Vahtera EM. Hemostatic factors and replacement of major blood loss with plasma-poor red cell concentrates. Anesth Analg. 1995;81:360–5. 7542432 10.1097/00000539-199508000-00026

[4] Fries D, Martini WZ. Role of fibrinogen in trauma-induced coagulopathy. Br J Anaesth. 2010;105:116–21. 20627882 10.1093/bja/aeq161

[5] Notani N, Miyazaki M, Kanezaki S, Ishihara T, Sakamoto T, Abe T, et al. Fibrinogen level on admission is a predictive marker of the need for massive blood transfusion after pelvic fracture. Am J Emerg Med. 2020;38:789–93. 31272757 10.1016/j.ajem.2019.06.043

[6] Nakamura Y, Ishikura H, Kushimoto S, Kiyomi F, Kato H, Sasaki J, et al. Fibrinogen level on admission is a predictor for massive transfusion in patients with severe blunt trauma: analyses of a retrospective multicentre observational study. Injury. 2017;48:674–9. 28122682 10.1016/j.injury.2017.01.031

[7] Inaba K, Karamanos E, Lustenberger T, Schöchl H, Shulman I, Nelson J, et al. Impact of fibrinogen levels on outcomes after acute injury in patients requiring a massive transfusion. J Am Coll Surg. 2013;216:290–7. 23211116 10.1016/j.jamcollsurg.2012.10.017

[8] Bouzat P, Ageron FX, Charbit J, Bobbia X, Deras P, Nugues JBD, et al. Modelling the association between fibrinogen concentration on admission and mortality in patients with massive transfusion after severe trauma: an analysis of a large regional database. Scand J Trauma Resusc Emerg Med. 2018;26:55. 29986757 10.1186/s13049-018-0523-0 PMC6038237

[9] Hagemo JS, Stanworth S, Juffermans NP, Brohi K, Cohen M, Johansson PI, et al. Prevalence, predictors and outcome of hypofibrinogenaemia in trauma: a multicentre observational study. Crit Care. 2014;18:R52. 24666991 10.1186/cc13798 PMC4056526

[10] Rourke C, Curry N, Khan S, Taylor R, Raza I, Davenport R, et al. Fibrinogen levels during trauma hemorrhage, response to replacement therapy, and association with patient outcomes. J Thromb Haemost. 2012;10:1342–51. 22519961 10.1111/j.1538-7836.2012.04752.x

[11] McQuilten ZK, Wood EM, Bailey M, Cameron PA, Cooper DJ. Fibrinogen is an independent predictor of mortality in major trauma patients: a five-year statewide cohort study. Injury. 2017;48:1074–81. 28190583 10.1016/j.injury.2016.11.021

[12] Rossaint R, Afshari A, Bouillon B, Cerny V, Cimpoesu D, Curry N, et al. The European guideline on management of major bleeding and coagulopathy following trauma: sixth edition. Crit Care. 2023;27:80. 36859355 10.1186/s13054-023-04327-7 PMC9977110

[13] Raykar NP, Makin J, Khajanchi M, Olayo B, Munoz Valencia A, Roy N, et al. Assessing the global burden of hemorrhage: the global blood supply, deficits, and potential solutions. SAGE Open Med. 2021;9:20503121211054995. 34790356 10.1177/20503121211054995 PMC8591638

[14] Škola J, Bílská M, Horáková M, Tégl V, Beneš J, Škulec R, et al. Shock Index for Early Detection of Low Plasma Fibrinogen in trauma: a prospective Observational Cohort Pilot Study. J Clin Med. 2023;12:1707. 36836242 10.3390/jcm12041707 PMC9966073

[15] Moore EE, Moore HB, Kornblith LZ, Neal MD, Hoffman M, Mutch NJ, et al. Trauma-induced coagulopathy. Nat Rev Dis Primers. 2021;7(1):30. 33927200 10.1038/s41572-021-00264-3 PMC9107773

[16] Kimura Y, Kimura S, Sumita S, Yamakage M. Predictors of hypofibrinogenaemia in blunt trauma patients on admission. J Anesth. 2015;29:242–8. 25112812 10.1007/s00540-014-1895-6

[17] Paydar S, Dalfardi B, Shayan Z, Shayan L, Saem J, Bolandparvaz S. Early predictive factors of hypofibrinogenaemia in Acute Trauma patients. J Emerg Trauma Shock. 2018;11:38–41. 29628667 10.4103/JETS.JETS_37_17 PMC5852914

[18] Schlimp CJ, Voelckel W, Inaba K, Maegele M, Ponschab M, Schöchl H. Estimation of plasma fibrinogen levels based on hemoglobin, base excess and Injury Severity score upon emergency room admission. Crit Care. 2013;17:R137. 23849249 10.1186/cc12816 PMC4056007

[19] Davenport R, Curry N, Fox EE, Thomas H, Lucas J, Evans A, et al. Early and empirical high-dose cryoprecipitate for Hemorrhage after traumatic Injury: the CRYOSTAT-2 Randomized Clinical Trial. JAMA. 2023;330:1882–91. 37824155 10.1001/jama.2023.21019 PMC10570921

[20] Gauss T, Campion S, Kerever S, Eurin M, Raux M, Harrois A, et al. Fibrinogen on admission in Trauma score: early prediction of low plasma fibrinogen concentrations in trauma patients. Eur J Anaesthesiol. 2018;35:25–32. 29120938 10.1097/EJA.0000000000000734

