# Correction: The utility of the prehospital shock index, age shock index, and modified shock index for predicting hypofibrinogenaemia in trauma patients: an observational retrospective study

**DOI:** 10.1007/s00068-024-02656-y

**Published:** 2024-09-03

**Authors:** Jihwan Moon, Sungwook Park

**Affiliations:** Department of Emergency Medicine, Pusan National University Hospital, 179, Gudeok-ro, Seo- Gu, Busan, 49241 Republic of Korea

**Correction: European Journal of Trauma and Emergency Surgery**

10.1007/s00068-024-02603-x

The original version of this article, published on 07 August 2024, unfortunately contained a mistake.

In Table 4 of this article, the third column was incorrectly labeled “Positive test probability (If a patient’s index is below the cut-off value)” but should read “Negative test probability (If a patient’s index is below the cut-off value)”.

The original article has been corrected.

